# Imagining Other People’s Experiences in a Person with Impaired Episodic Memory: The Role of Personal Familiarity

**DOI:** 10.3389/fpsyg.2012.00588

**Published:** 2013-01-24

**Authors:** Jennifer S. Rabin, Nicole Carson, Asaf Gilboa, Donald T. Stuss, R. Shayna Rosenbaum

**Affiliations:** ^1^Department of Psychology, York UniversityToronto, ON, Canada; ^2^Rotman Research Institute, Baycrest HospitalToronto, ON, Canada; ^3^Department of Psychology, University of TorontoToronto, ON, Canada; ^4^The Heart and Stroke Foundation, Centre for Stroke RecoveryToronto, ON, Canada; ^5^Ontario Brain InstituteToronto, ON, Canada; ^6^Division of Neurology, Department of Medicine, University of TorontoToronto, ON, Canada; ^7^Rehabilitation Sciences, University of TorontoToronto, ON, Canada

**Keywords:** episodic memory, theory of mind, hippocampus, amnesia, social cognition

## Abstract

Difficulties remembering one’s own experiences via episodic memory may affect the ability to imagine other people’s experiences during theory of mind (ToM). Previous work shows that the same set of brain regions recruited during tests of episodic memory and future imagining are also engaged during standard laboratory tests of ToM. However, hippocampal amnesic patients who show deficits in past and future thinking, show intact performance on ToM tests, which involve unknown people or fictional characters. Here we present data from a developmental amnesic person (H.C.) and a group of demographically matched controls, who were tested on a naturalistic test of ToM that involved describing other people’s experiences in response to photos of personally familiar others (“pToM” condition) and unfamiliar others (“ToM” condition). We also included a condition that involved recollecting past experiences in response to personal photos (“EM” condition). Narratives were scored using an adapted Autobiographical Interview scoring procedure. Due to the visually rich stimuli, internal details were further classified as either descriptive (i.e., details that describe the visual content of the photo) or elaborative (i.e., details that go beyond what is visually depicted in the photo). Relative to controls, H.C. generated significantly fewer elaborative details in response to the pToM and EM photos and an equivalent number of elaborative details in response to the ToM photos. These data converge with previous neuroimaging results showing that the brain regions underlying pToM and episodic memory overlap to a greater extent than those supporting ToM. Taken together, these results suggest that detailed episodic representations supported by the hippocampus may be pivotal for imagining the experiences of personally familiar, but not unfamiliar, others.

## Introduction

Amnesia following damage to the hippocampus has been characterized by impaired episodic memory for personally experienced events. However, there is growing evidence that other, non-mnemonic processes may be compromised in amnesia as well. These findings have led researchers to suggest a broader role for the hippocampus and episodic memory that goes beyond recalling past personal experiences. Much of this work has focused on the idea that episodic memory is necessary for imagining possible future scenarios (Tulving, 1985; Klein et al., 2002; Okuda et al., 2003; Rosenbaum et al., 2005; Addis et al., 2007; Szpunar et al., 2007; Andelman et al., 2010), whereas much less attention has been paid to the role that episodic memory plays in social behavior. In the current study, we examined if, and under what conditions, the ability to remember and imagine one’s own experiences serves a social function in facilitating the ability to imagine other people’s experiences.

An impressive body of research has shown that episodic memory, supported by the hippocampus, is closely related to the ability to imagine one’s own personal future. Amnesic individuals with hippocampal damage who are unable to recollect past events also have difficulty imagining themselves in future events (Tulving, 1985; Klein et al., 2002; Rosenbaum et al., 2005; Andelman et al., 2010). Consistent with this finding, neuroimaging studies have revealed that both abilities recruit a similar set of brain regions that include the hippocampus and adjacent medial temporal lobe (MTL) regions as well as medial frontal, medial parietal, and lateral temporal cortex (Okuda et al., 2003; Addis et al., 2007; Szpunar et al., 2007). Some of these studies have included a control condition in which participants are asked to imagine the experiences of an “average” person or a famous person, which appears to engage regions within the MTL as well, albeit to a lesser extent (Szpunar et al., 2007; see also Gilboa et al., 2004). However, it may be the case that episodic memory and associated MTL function play an important role in imagining other people’s experiences, as suggested by qualitative reviews and meta-analyses of the neuroimaging literature. These studies show that the same set of brain regions activated during tests of episodic memory and future imagining are also engaged during standard tests of theory of mind (ToM; Buckner and Carroll, 2007; Hassabis and Maguire, 2007; Spreng et al., 2009).

In addition to an overlapping set of brain regions, episodic memory, future imagining, and ToM emerge close in time in ontogenetic development (Perner and Ruffman, 1995; Atance and O’Neil, 2001; Perner et al., 2007) and tend to be impaired in patients with schizophrenia (Corcoran and Frith, 2003; D’Argembeau et al., 2008) and high functioning autism and Asperger’s syndrome (Adler et al., 2010; Lind and Bowler, 2010). These findings lend support to an influential theoretical perspective that individuals draw on past experiences via episodic memory to simulate future personal experiences and to imagine other people’s experiences during ToM (Gordon, 1986; Goldman, 1992; Corcoran, 2000, 2001; Gallagher and Frith, 2003; Buckner and Carroll, 2007; Schacter and Addis, 2007; Spreng and Mar, 2012). However, work with hippocampal amnesic patients shows preserved performance on standard tests of ToM despite impaired episodic memory and future imagining (Rosenbaum et al., 2007; Rabin et al., 2012). Standard ToM tests included in these studies ranged from predicting a character’s false belief (Stone et al., 1998) and identifying a faux pas (Stone et al., 1998) based on narratives, to inferring others’ thoughts and emotions based on viewing the eye region of faces (Baron-Cohen et al., 2001). The amnesic patients’ successful performance on these tests may have been achieved via semantic memory, which remains relatively intact in these patients (Rosenbaum et al., 2007). This might include reliance on social knowledge of the average person’s thoughts, feelings, and intentions in different circumstances (Lieberman, 2012).

More recent neuroimaging studies have directly compared episodic memory with ToM in the same individuals using more naturalistic stimuli (Rabin et al., 2010; Spreng and Grady, 2010; St. Jacques et al., 2011; see also Gilboa et al., 2004; Szpunar et al., 2007). These studies revealed that relative to recalling past episodes, imagining the experiences of other people elicited less activity within MTL and midline regions. However, the “other” targets in these studies were not intimately known by participants (i.e., strangers or public figures). It is possible that when the target person is personally known, shared past experiences can influence participants’ current social processing. Indeed, knowing someone for a long period of time and observing that person’s behavior in different situations provides a rich source of information from which one can draw when imagining his/her mental states in specific situations. Consistent with this idea, Rabin and Rosenbaum (2012) recently showed that imagining the experiences of personally familiar versus unfamiliar others preferentially engaged regions known to support episodic memory, suggesting a strategy of relying on past personal experiences when the target person is personally known. In another study, Krienen et al. (2010) focused exclusively on midline frontal regions and found greater anterior medial prefrontal cortex and rostral anterior cingulate cortex activity for judgments relating to participants’ friends versus strangers. In fact, participants in that study indicated that they relied on a specific memory or anecdote significantly more often for judgments relating to friends than strangers. Perry et al. (2011) showed that hippocampal activity during judgments of others’ emotional states was specific to conditions in which the protagonist was deemed similar to the self and when the event had occurred in the participant’s own life. Taken together, these studies suggest that episodic memory may serve a social role in imagining other people’s experiences, but only when intimacy or closeness exists between the participant and the perceived other.

In the current study, we test the idea that episodic memory is necessary for imagining events from the perspective of personally known others. One way to address this question is to assess whether a person with hippocampal amnesia and impaired episodic memory is able to imagine events experienced by well-known others, including reconstructing others’ thoughts and feelings. Here, we test H.C., a unique young woman with normal intellectual function despite impaired development of her episodic memory due to selective hippocampal damage 1 week after birth (Vargha-Khadem et al., 2003; Rosenbaum et al., 2011; see also Kwan et al., 2010; Hurley et al., 2011). Importantly, as was the case for the adult-onset hippocampal amnesic cases described above, we recently found that H.C.’s performance on a wide range of standard ToM tests was indistinguishable from that of controls (Rabin et al., 2012). We believe that her preserved ToM performance is due to reliance on her semantic memory and general knowledge abilities, which remain relatively intact (Rabin et al., 2012). In the current study we employed a naturalistic test of ToM that involved describing the experiences of other people in response to photos of personally known others (i.e., relatives and close friends; “pToM” condition) and unknown others (“ToM” condition) engaging in specific events. We also included a condition that involved recollecting past experiences in response to personal photos (“EM” condition). This naturalistic task was selected because it is less constrained than most standard tests of ToM and therefore better captures ToM as it occurs in everyday life. Findings of impaired pToM that parallel H.C.’s episodic memory deficit would suggest that pToM relies on episodic memory or that a common process mediates both abilities. Alternatively, it may be the case that intact aspects of H.C.’s semantic memory are sufficient to support mental state inferences involving pToM and ToM, and therefore H.C. would show intact performance on both tasks, similar to her performance on standard ToM tests.

## Materials and Methods

### Participants

H.C. is a right-handed woman who was 20 years old at the time of testing. A second testing session was performed when H.C. was 23 years old for reliability purposes. She was born prematurely and suffered hypoxic damage, which led to reduced bilateral hippocampal volume by approximately 50% relative to healthy controls (Vargha-Khadem et al., 2003; Hurley et al., 2011; see Rosenbaum et al., 2011 for a detailed neuropsychological profile). H.C.’s compromised bilateral hippocampal development appears to have precluded normal development of her episodic memory. Her impairment affects her personal and public event memory more than her personal and general semantic memory (Rosenbaum et al., 2011), which is consistent with other developmental amnesic cases (Gadian et al., 2000). H.C. successfully graduated from a mainstream high school and completed 1 year of technical college. At the time of the first testing session, she was enrolled in a post-secondary culinary program but withdrew after 1 year. H.C. has formed a normal number of close relationships (Davidson et al., 2012, this issue) and was engaged to be married at the second time of testing.

H.C.’s performance on all measures was compared with that of 18 right-handed, healthy women with no reported history of neurological or psychiatric illness (mean age = 19.4, SD = 1.3; mean education = 13.3, SD = 1.1). All participants gave informed written consent in accordance with the ethics review boards at York University and Baycrest. Participants received monetary compensation for their time.

### Stimuli

We employed a novel, naturalistic test of ToM that involved describing others’ thoughts and feelings in response to photos of personally familiar others (“pToM” condition) and unfamiliar others (“ToM” condition) engaging in specific events. We also included a condition that involved recollecting past experiences in response to personal photos (“EM” condition; Rabin and Rosenbaum, 2012).

The pToM condition involving personally known others consisted of 15 photos depicting specific events that had been experienced by family members and close friends but not by the participant him/herself. The ToM condition involving unfamiliar others consisted of 15 photos depicting strangers engaged in specific events. The EM condition consisted of 15 personal family photos of events that took place within the past 1–5 years. H.C. and 13 of the 18 control participants appeared in each EM photo to help verify that the participant personally experienced the event. Analyses confirmed that the presence or absence of the control participants in the EM photos did not affect the behavioral results (i.e., average number of internal details did not differ, *t*(16) = −0.47, *p* = 0.64). The pToM and EM photos were collected by a relative or close friend of each participant, whereas the ToM photos were collected by the experimenter. Themes were similar across the three conditions (e.g., birthday party, picnic, vacation) and included both indoor and outdoor scenes. All photos were resized and converted to gray scale.

### Task

H.C. and the control participants were scanned with fMRI while performing the family photos task (fMRI data not reported here). Stimuli were presented in blocks and each block contained five photos from one of the three conditions. There were three blocks for each condition (for a total of nine blocks) and these were presented in pseudorandom order. At the beginning of each block, participants viewed a set of instructions that corresponded to one of the three conditions (i.e., pToM, ToM, or EM). Each photo was presented for 20 s and was followed by three rating scales (see below).

In the pToM and ToM conditions, participants were presented with photos of other people and asked to generate a novel event for each photo while focusing on what one person in the photo might have been thinking and feeling at the time. In order to distinguish imagining from remembering, participants were specifically instructed not to draw on past experiences when generating these events. In the EM condition, participants were presented with their own photos and asked to recollect the event depicted in each photo in as much detail as possible. They were told to focus on what they were thinking and feeling at the time.

Following the presentation of each photo, participants rated the events they imagined/recollected on a number of dimensions. Three ratings scales were presented after each photo. The first rating scale differed for the pToM/ToM and EM events. The pToM and ToM events were rated for likeness to an actual memory (1 = exactly like a memory … 4 = nothing like a memory), whereas the EM events were rated on the extent to which the events were recollected (1 = don’t know event; 2 = familiar with event; 3 = remember event; Gardiner et al., 1998; Tulving, 1985). Participants were instructed to select “remember” if the event was specific to a time and place and they could re-experience it, to select “familiar with event” if the event was familiar to them, but they could not recall any specific contextual or other experiential details associated with the event, and to select “don’t know event” if they were unable to recall any aspect of the event. The next two ratings scales were employed for all conditions. One scale assessed the amount of detail generated for each event (1 = not vivid … 4 = very vivid) and the other scale assessed the spatial coherence of each event (contiguousness of the spatial context: 1 = fragmented scenes … 4 = continuous scene; Hassabis et al., 2007; not reported in the current study).

Prior to the scan, a short training session was provided to ensure that participants fully understood the task instructions. The photos used in the training session were not used during the scan.

Immediately following the scan, participants took part in an interview in which they viewed the same photos that had been presented in the scanner. Participants were asked to think back to the events they generated in the scanner and to rate each event on the same three scales that were presented in the scanner. The photos with the highest vividness ratings (approximately two-thirds of all photos) were selected for a semi-structured interview in which participants described the events as they had been imagined/recollected in the scanner.^1^ High vividness ratings were taken to suggest that participants were indeed imagining or recollecting the events. There was no time limit for participants to describe the events, and participants continued with their descriptions until they came to a natural ending point. The examiner then provided a single, standardized probe to elicit additional details (e.g., “Can you tell me anything else?”). The events were recorded and transcribed for scoring.

Control participants were tested on the family photos paradigm once whereas H.C. was tested on the paradigm on two separate occasions for reliability purposes. However, the EM events that were included during H.C.’s first testing session were excluded because we subsequently learned that she frequently views and rehearses the events depicted in these photos.

### Scoring

Narratives were scored using an adapted Autobiographical Interview scoring procedure described by Levine et al. (2002). The pToM, ToM, and EM events were first segmented into distinct details, which were classified as internal (including event-specific, temporal, perceptual, spatial, and thought/emotion details) or external (i.e., semantic facts that were irrelevant to the central event, repetitions, and metacognitive statements). Given the use of visually rich photos as cues, we wanted to ensure that participants’ performance was not inflated due to merely describing the details depicted in the photos. Therefore, internal details were further classified as either descriptive (i.e., details that describe the visual content of the photo) or elaborative (i.e., details that go beyond what is visually depicted in the photo; see Table 1 for scoring criteria).

**Table 1:** **Classification of descriptive versus elaborative details**.

Type of detail	Descriptive details	Elaborative details
Action	Any detail referring to an action that is depicted in the photo (e.g., sitting, walking, standing, posing for the photo)	Any detail describing an action that is not obvious from the photo
Character	Any detail explaining who the people are in the photo (only for the pToM and EM conditions)	Any detail describing who the people are or any detail that refers to the relationship(s) between the people depicted in the photo (only for the ToM condition)
Temporal	N/A	Any detail referring to a specific time period (e.g., year, season, month, date, day of week)
Perceptual	Perceptual details that are depicted in the photo (e.g., big crowd of people, candles everywhere). Describing or naming an object, monument or statue that is depicted in the photo (e.g., Statue of Liberty)	Perceptual details that are not visible in the photo
Emotion/thought	Any detail describing a facial expression (e.g., smiling, frowning)	Any detail describing an emotion or mental state (e.g., happy, sad, tired)
Spatial/Place	Any detail describing a location (e.g., country, city, street, location within a room) that can be inferred from information presented in the photo (e.g., sign)	Any detail describing a location (e.g., country, city, street, location within a room) that is not apparent from information depicted in the photo

Scoring of the narratives was conducted by a trained rater who achieved high interrater reliability on the Autobiographical Interview using a standard set of previously scored memories (see Levine et al., 2002). Interrater reliability was also calculated for the elaborative and descriptive details based on criteria developed by JSR. Intraclass correlation analyses indicated high agreement among scorers for pToM (Cronbach’s α = 0.994), ToM (Cronbach’s α = 0.992), and EM events (Cronbach’s α = 0.994).

Data were analyzed using a modified *t*-test procedure, which compares test scores of a single patient to that of a small control sample (Crawford and Howell, 1998). Two-tailed *t*-tests were used to compare H.C.’s performance with that of controls on the pToM and ToM conditions, whereas a one-tailed *t*-test was used for the EM condition given *a priori* hypotheses regarding H.C.’s episodic memory performance.

## Results

As mentioned above, H.C. was tested on two separate occasions. For completeness, we report the data separately for the two testing sessions. Each control participant contributed an average of 8.9 pToM events (SD = 0.72), 9.1 ToM events (SD = 0.9), and 9.3 EM events (SD = 0.49) to the analyses. In session 1, H.C. contributed 7 pToM events and 9 ToM events to the analyses. In session 2, H.C. contributed 15 pToM events, 12 ToM events, and 18 EM events to the analyses.

### Phenomenology of the pToM, ToM, and EM events

We entered participants’ post-scan ratings into the analyses (as opposed to the within-scanner ratings) as these were believed to better correspond with the events participants described during the post-scan interview. Table 2 presents participants’ phenomenological ratings of the pToM, ToM, and EM events. In terms of vividness, H.C. rated the pToM events in session 1 as less vivid than controls, *t*(17) = −2.68, *p* = 0.02; there was no difference for the pToM events in session 2, *t*(17) = −0.73, *p* = 0.48. With respect to the ToM events, vividness did not differ between H.C. and controls for session 1, *t*(17) = −0.97, *p* = 0.34, or session 2, *t*(17) = −1.46, *p* = 0.16. For the EM events, H.C.’s ratings were significantly less vivid than that of controls, *t*(17) = −3.89, *p* = 0.0006. In terms of the ratings assessing likeness to an actual memory, no significant differences emerged between H.C. and controls for the pToM and ToM events in session 1 or session 2 [pToM session 1 and session 2, *t*(17) = −1.56, *p* = 0.14, and *t*(17) = −0.38, *p* = 0.70, respectively, and ToM session 1 and session 2, *t*(17) = −0.58, *p* = 0.57, and *t*(17) = −0.58, *p* = 0.57, respectively]. Finally, as expected, H.C.’s ratings relating to the recollection of EM events were significantly lower than that of controls, *t*(17) = −9.73, *p* < 0.00001.

**Table 2:** **Phenomenological qualities of the generated pToM, ToM, and EM events**.

	pToM	ToM	EM
Vividness			
H.C. session 1	2.1*	2.7	–
H.C. session 2	2.9	2.5	2.8*
Controls	3.2 (0.4)	3.1 (0.4)	3.6 (0.2)
Remember/know			
H.C. session 1	–	–	
H.C. session 2	–	–	2.6*
Controls	–	–	3.0 (0.04)
Similar to a Memory			
H.C. session 1	2.7	3.3	–
H.C. session 2	3.3	3.3	–
Controls	3.5 (0.5)	3.6 (0.5)	–

Standard deviations are given in parentheses; pToM, personal theory of mind; ToM, theory of mind; EM, episodic memory; *p < 0.05.

### Adapted autobiographical interview

Given the use of visually rich photos as cues, we were most interested in the elaborative details that participants generated. We analyzed the data in two ways. First, we compared the average number of elaborative details H.C. and controls produced in response to each pToM, ToM, and EM event. These absolute numbers, however, are confounded by participants’ total verbal output. To overcome this issue, we also calculated the proportion of elaborative-to-total internal details, which provides an index of the weight given to descriptive versus elaborative details.

**Figure 1:**
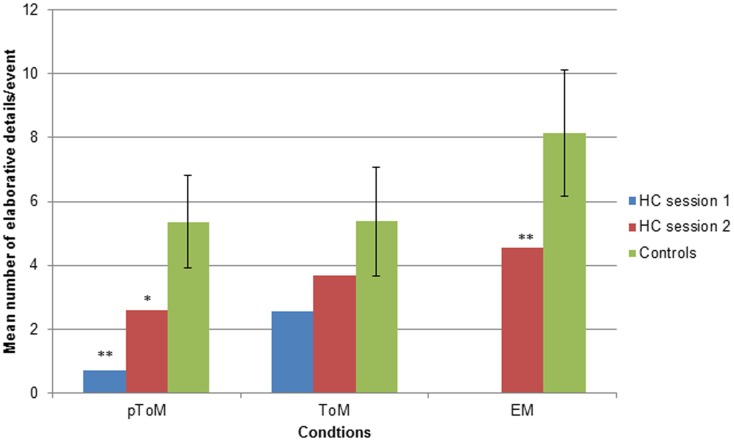
The mean number of elaborative details provided by H.C. and controls in response to each pToM, ToM, and EM event, *p < 0.08; **p < 0.05. Error bars indicate standard deviations.

The mean number of elaborative details produced by participants in response to each pToM, ToM, and EM event is presented in Figure 1,^2^. In response to the pToM events, H.C. produced significantly fewer elaborative details than controls during session 1, *t*(17) = −3.1, *p* = 0.007, and there was a trend toward impaired performance during session 2, *t*(17) = −1.8, *p* = 0.08. In terms of the ToM events, no significant group difference emerged for session 1, *t*(17) = −1.6, *p* = 0.13, or session 2, *t*(17) = −0.98, *p* = 0.34. With respect to the EM events, as expected, H.C. produced significantly fewer elaborative details than controls, *t*(17) = −1.78, *p* = 0.047^3^.

**Figure 2:**
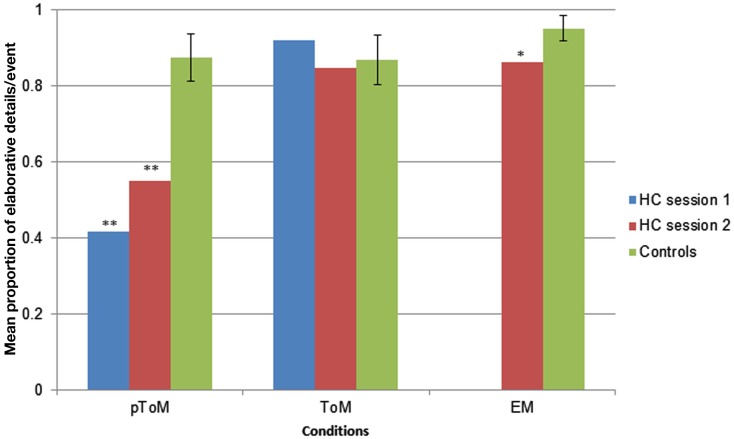
The mean proportion of elaborative-to-total-number of internal details provided by H.C. and controls in response to each pToM, ToM, and EM event, *p < 0.01; **p < 0.0001. Error bars indicate standard deviations.

The mean proportion of elaborative-to-total-internal details produced by participants in response to each pToM, ToM, and EM event is presented in Figure 2. Analyses revealed that H.C. produced a lower proportion of elaborative details (and therefore a greater number of descriptive details) than controls in response to the pToM events during both session 1, *t*(17) = −7.0, *p* < 0.00001 and session 2, *t*(17) = −4.99, *p* = 0.0001. In contrast, H.C. and controls produced an equivalent proportion of elaborative details in response to the ToM events during both session 1, *t*(17) = 0.77, *p* = 0.45, and session 2, *t*(17) = −0.32, *p* = 0.75. Consistent with our predictions, H.C. generated a lower proportion of elaborative details relative to controls in response to the EM events, *t*(17) = −2.57, *p* = 0.01.

## Discussion

H.C., a developmental amnesic person with bilateral hippocampal damage, was impaired at imagining the experiences of personally known others, which resembles her compromised ability to recall past experiences via episodic memory. These impairments stand in contrast to her preserved ability to imagine the experiences of unknown others. This pattern of results held whether we analyzed the average number of elaborative details (i.e., details that go beyond what is visually depicted in the photo) or the proportion of elaborative-to-total-internal details in order to control for verbal output. These results bolster the finding that different neural and cognitive mechanisms support thinking about personally known versus unknown others and that the former may depend on processes mediated by the hippocampus and episodic memory.

The idea that individuals rely on past personal experiences to infer and simulate another’s mental state has been suggested by philosophers and cognitive neuroscientists alike (Corcoran, 2000, 2001; Gallagher and Frith, 2003; Buckner and Carroll, 2007; Spreng and Mar, 2012). However, the current findings indicate that reliance on past personal experiences may be pivotal only when imagining the experiences of personally known others. Indeed, knowing an individual for a long period of time and observing that person’s behavior in different situations provides a rich source of information from which one can draw when imagining his/her mental states in various situations. Consistent with this interpretation, Krienen et al. (2010) showed that participants reported that they relied on a specific memory or anecdote significantly more often when making judgments relating to friends relative to strangers. In another study, Ciaramelli et al. (submitted) found that participants’ level of empathy for a familiar character was modulated by the retrieval of previous episodes involving that character. Furthermore, using the same family photos paradigm employed in the current study, we (Rabin and Rosenbaum, 2012) showed that the pattern of neural activity supporting pToM shares more in common with episodic memory than with ToM. Notably, the greatest degree of neural overlap between pToM and episodic memory was observed within midline regions, including the hippocampus and related MTL structures, regions traditionally associated with the recollection of past events.

Reliance on past personal experiences to infer familiar others’ mental states may occur with or without one’s intention or awareness. There is accumulating evidence that episodic memory supported by the hippocampus can rapidly and automatically influence performance on non-mnemonic tasks (Westmacott and Moscovitch, 2003; Westmacott et al., 2004; Moscovitch, 2008; Ryan et al., 2008; Greenberg et al., 2009; Sheldon and Moscovitch, 2010). Gobbini and Haxby (2007) suggest that the mere perception of a familiar individual is associated with the spontaneous retrieval of personal knowledge about that individual (i.e., personal traits, attitudes, biographical facts, and episodic memories), which in turn may help to better understand and predict what the familiar other is thinking and/or feeling. These automatic processes may have been at play in the current study given that participants were instructed not to refer to past episodes when generating the pToM and ToM events. It is possible that participants engaged in inhibitory processes to help overcome the prepotent tendency to rely on past memories. Alternatively, other memory regulation processes, such as thought substitution (Benoit and Anderson, 2012) may have been employed.

Another possible explanation for H.C.’s corresponding impairment in both episodic memory and pToM may relate to a deficit in (re)constructing specific episodes. Evidence from neuroimaging studies suggests that imagining specific versus general past and future events elicits greater activity within the hippocampus (Addis et al., 2011; Ford et al., 2011), likely due to the greater relational processing that is required for the former (Addis et al., 2011). Several researchers have argued that individuals are more likely to imagine close others with greater specificity relative to unknown others. In contrast, unknown others are typically represented in more generic and abstract terms (Liviatan et al., 2008; Lieberman, 2012). This may be because we possess idiosyncratic theories about close others’ personalities that enable us to richly imagine how well-known others would respond in various scenarios (see Lieberman, 2012). Therefore H.C.’s difficulty in generating specific details may account for her poor performance on the episodic memory and pToM tasks.

It may be the case that for the pToM events H.C. attempted to rely on a strategy that is optimal for people who are able to conjure up contextual and specific details rather than relying on a strategy that would be advantageous for her. Like controls, H.C. may have been engaging in inhibitory processes of past events when generating the pToM and ToM events. However, because her episodic recollection is impaired, she may have generalized this instruction to personal semantic information, which would have likely helped her to generate additional details for the pToM events. It is possible that if she had been probed in a manner that enabled her to draw more effectively on her intact personal or social semantic memory, she may have performed better on the pToM task. Indeed, different methods of cuing can differentially affect task performance. H.C., for instance, was impaired at imagining herself in future episodes when provided with a specific cue word (e.g., “coffee”; Kwan et al., 2010) but showed intact performance when a more general and non-specific cue was provided (e.g., “Imagine something you will be doing this weekend”; Hurley et al., 2011; see also Cooper et al., 2011).

The corresponding deficit that emerged in episodic memory and pToM is unlikely to be due to a deficit in narrative construction, given that H.C. had no difficulty constructing narratives in response to the ToM events. This pattern of results is consistent with those from a recent study showing that the ability to generate a detailed narrative is preserved in adult-onset amnesia (Race et al., 2011; but see Rosenbaum et al., 2009). Although the patients in the study by Race and colleagues produced impoverished descriptions of past and future events, they showed intact performance when asked to tell a story in response to pictures depicting fictional characters in various scenes. It is important to note that while their participants were instructed to generate a story rather than to report what was literally depicted in the picture, to our knowledge, the authors did not examine whether participants adhered to this instruction. In the current study, when examining the extent to which participants relied on the visual content of the photos to generate details, we found that approximately half of the details H.C. produced for the pToM events consisted of descriptive details (vs. 12.5% for controls). The current findings highlight the importance of examining descriptive versus elaborative details when rich visual cues are used.

H.C.’s impairment in episodic memory and pToM contrasts with her preserved ability to imagine the experiences of unknown others during ToM. The latter finding is consistent with her intact performance on a wide range of standard ToM tests that employ strangers or fictional characters as targets (Rabin et al., 2012; see also Rosenbaum et al., 2007). Imagining the experiences of unfamiliar others may be achieved by relying on social semantic memory, which remains relatively intact in H.C. This might include reliance on generic representations about how the average person is expected to think and feel in a given situation (Lieberman, 2012). Generic representations are likely based on routines or schemas that are already bound together and therefore require minimal relational processing. Recent fMRI findings from our laboratory (Rabin and Rosenbaum, 2012), support this interpretation. Using the same family photos paradigm, we recently showed that relative to pToM, ToM involving unfamiliar others elicited greater activity in lateral regions known to be associated with accessing semantic knowledge (Martin and Chao, 2001). Taken together, these data further corroborate the notion that episodic memory may be needed for social cognition, but that its role may be specific to imagining the experiences of personally known, and not unknown, others.

The use of an open-ended ToM task allowed us to gain insight into possible compensatory strategies that H.C. employed when taking the perspective of another person. We found that H.C. generated a significantly greater proportion of descriptive details in response to the pToM photos than did controls, suggesting that she relied more heavily on the visual information depicted in the photos to imagine the experiences of well-known others. This may have included relying on the familiar other’s facial expression, body language, and/or the relative spatial relations between people. This strategy may serve her well in social settings when external cues are readily available but may fail when cues are absent or when situations are complex and require the integration of information from the past and present.

**Figure 3:**
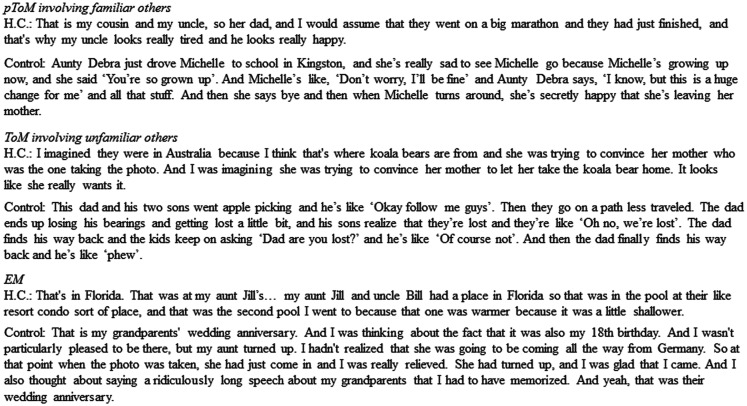
**Representative samples of the pToM, ToM, and EM narratives provided by H.C. and a control participant**.

H.C.’s performance on the pToM condition was not at floor indicating that her ability to imagine the experiences of personally familiar others is not obliterated. In fact, approximately 50% of the details she generated in response to the pToM events were elaborative details (i.e., details that go beyond what is visually depicted in the photo). However, upon closer examination, even the qualitative nature of the elaborative details she generated differed from that of controls. Specifically, H.C.’s responses tended to reflect more basic emotional states that could be inferred from the visual features of the photo, such as “they’re both really excited” or “she looks really happy.” In contrast, control participants typically provided more complex mental state inferences such as “they were probably afraid but they are trying to look cool” and “her mother was pleased that her daughter was having so much fun” (see Figure 3 for narrative samples).

H.C. generated a greater number of elaborative details in response to the pToM and ToM events during session 2 relative to session 1. It is important to note, however, that the overall pattern remained consistent across the two testing sessions in that, in both cases, H.C. produced fewer elaborative details for the pToM versus ToM events. It is possible that the difference across testing sessions reflects a practice effect resulting from experience with narrative generation. Although our two testing sessions took place 3 years apart, H.C. participated in several other studies that required her to generate detailed narratives in the interim (Kwan et al., 2010, 2011; Hurley et al., 2011). In fact, within these other studies, H.C. showed improved performance on tests of future imagining across testing sessions (Kwan et al., 2010; Hurley et al., 2011). A related explanation for H.C.’s inflated scores during session 2 is that she may have learned to use a more effective strategy that enabled her to generate a greater number of details.

In the current study, we attempted to control for vividness by only including the pToM, ToM, and EM events with the highest vividness ratings in our analyses. Nevertheless, analyses revealed that H.C. rated the pToM events in session 1 and the EM events in session 2 as less vivid than controls. In addition, we cannot rule out that other factors, such as personal significance, differed between H.C. and controls.

In conclusion, using an ecologically valid test of ToM, we formally document that episodic memory supported by the hippocampus may be pivotal for imagining the experiences of personally familiar, but not unfamiliar, others. The current findings complement recent fMRI data and suggest that when imagining other people’s experiences individuals are more likely to rely on episodic memory when the target person is personally familiar and on general social semantic memory when the target person is unknown (Rabin and Rosenbaum, 2012). Continued research with H.C. and other amnesic individuals, particularly those that acquire damage later in life, is needed to better understand the role that episodic memory plays in this and other aspects of social cognition.

## Conflict of Interest Statement

The authors declare that the research was conducted in the absence of any commercial or financial relationships that could be construed as a potential conflict of interest.

## Footnotes

^1^During session 2, H.C. was interviewed on all of the photos presented during the scan as well as eight additional EM events, which were not presented in the scanner, in order to increase power.

^2^Due to the small number of elaborative details produced for each event, we were unable to make meaningful comparisons when the details were further divided into the internal detail categories (i.e., event, place, time, perceptual, thought/emotion).

^3^We did not confirm the accuracy of participants’ reported memories. However, anecdotal evidence provided by H.C.’s family suggests that she tends to fill in memory gaps. Therefore, H.C.’s EM scores are likely an overestimate of her episodic memory capabilities.
